# Case Report: Potential Predictive Value of MMR/MSI Status and PD-1 Expression in Immunotherapy for Urothelial Carcinoma

**DOI:** 10.3389/pore.2022.1610638

**Published:** 2022-10-21

**Authors:** Yu-Ting Ma, Yan Li, Li Yan, Fang Hua, Dong-Guan Wang, Guo-Ying Xu, Hong-Lan Yang, Ying-Jie Xue, Ye-Jun Qin, Dan Sha, Hao Ning, Miao-Qing Zhao, Zhi-Gang Yao

**Affiliations:** ^1^ Department of Pathology, Shandong Provincial Hospital Affiliated to Shandong First Medical University, Jinan, China; ^2^ Department of Oncology, Dongying City People’s Hospital, Dongying, China; ^3^ Department of Pathology, Dongying City People’s Hospital, Dongying, China; ^4^ Department of Microbiology and Immunology, Tulane University, New Orleans, LA, United States; ^5^ Department of Urology Surgery, Dongying Hospital of Traditional Chinese Medicine, Dongying, China; ^6^ Department of Oncology, Shandong Provincial Hospital Affiliated to Shandong First Medical University, Jinan, China; ^7^ Department of Urology, Shandong Provincial Hospital Affiliated to Shandong First Medical University, Jinan, China; ^8^ Department of Pathology, Shandong Cancer Hospital and Institute, Shandong First Medical University, Shandong Academy of Medical Sciences, Jinan, China

**Keywords:** immunotherapy, microsatellite instability, immune checkpoint inhibitors, urothelial carcinoma, lynch syndrome, PD-1/PD-L1

## Abstract

Immune checkpoint inhibitors (ICIs) have shown encouraging outcomes against Lynch syndrome (LS)-associated colorectal cancer (CRC) and endometrial cancer with mismatch repair deficient/microsatellite instability–high (dMMR/MSI-H). However, there is as yet no clarity on the safety and efficacy of immunotherapy combined with chemotherapy in LS-associated urothelial carcinoma (UC). Here, we report a patient with recurrent and metastatic LS-associated UC who achieved sustained response to programmed death protein 1 (PD-1) inhibitor combined with chemotherapy over 31 months, during which the side effects of immunotherapy could be controlled and managed. Our findings indicate that the dMMR/MSI status and PD-1 expression in UC may have potential predictive value for the response to PD-1-targeted immunotherapy. Our case supports the inclusion of such combination and/or monotherapy for UC in clinical studies and using dMMR/MSI status and PD-1 expression as potential predictive biomarkers for assessment of the therapeutic response.

## Introduction

Urothelial carcinoma (UC) is a common malignant tumor that occurs in the bladder or upper urinary tract. In recent decades, surgery and combination chemotherapy demonstrate the improved efficacy of UC. However, a proportion of patients with advanced and metastatic UC after first-line and second-line treatment have a particularly poor prognosis [1, 2]. Several ongoing clinical trials in UC are exploring the potential survival benefit from immune checkpoint inhibitors (ICIs) targeting the programmed death protein 1 (PD1)/PD-L1 (ligand) pathway [3]. The utility of immunotherapy is expected to shape the future treatment of UC.

As one of the most common hereditary cancer predisposition syndromes, Lynch syndrome (LS) is caused by germline mutations in the DNA mismatch repair (MMR) genes, resulting in deficient MMR (dMMR). LS is associated with a markedly increased lifetime risk of colorectal cancer (CRC); endometrial cancer; cancers of the urothelial tract, stomach, ovary, pancreas, biliary tract; and sebaceous neoplasms of the skin. Recently, many studies have demonstrated that PD1/PD-L1 blockade therapies have profound implications for the medical management of LS-associated advanced CRC and endometrial cancer [4, 5]. However, reports of successful treatment of metastatic LS-associated UC using ICIs are limited. Here, we present a patient with LS who developed recurrent UC with lung/bone metastasis and displayed a favorable and sustained response to PD-1 inhibitors combined with chemotherapy. An additional observation worth discussing is the potential predictive biomarkers of clinical response to PD-1-targeted immunotherapy in UC.

## Case Report

### Clinical Course

A 62-year-old man suffering from gross hematuria was referred to Dongying People’s Hospital in March 2017. He was diagnosed with ureteral cancer and synchronous bladder cancer based on images, and underwent nephroureterectomy and transurethral resection of bladder tumor (TURBT). Invasive high-grade UC was confirmed on pathology examination. Additional instillation of pirarubicin (30 mg) weekly for 8 weeks was done after the operation. After that, three TURBT procedures were performed for recurrent bladder UC between May and November of 2017, during which intravesical instillations of Bacillus Calmette–Guérin (BCG) were carried out consistently.

**FIGURE 1:**
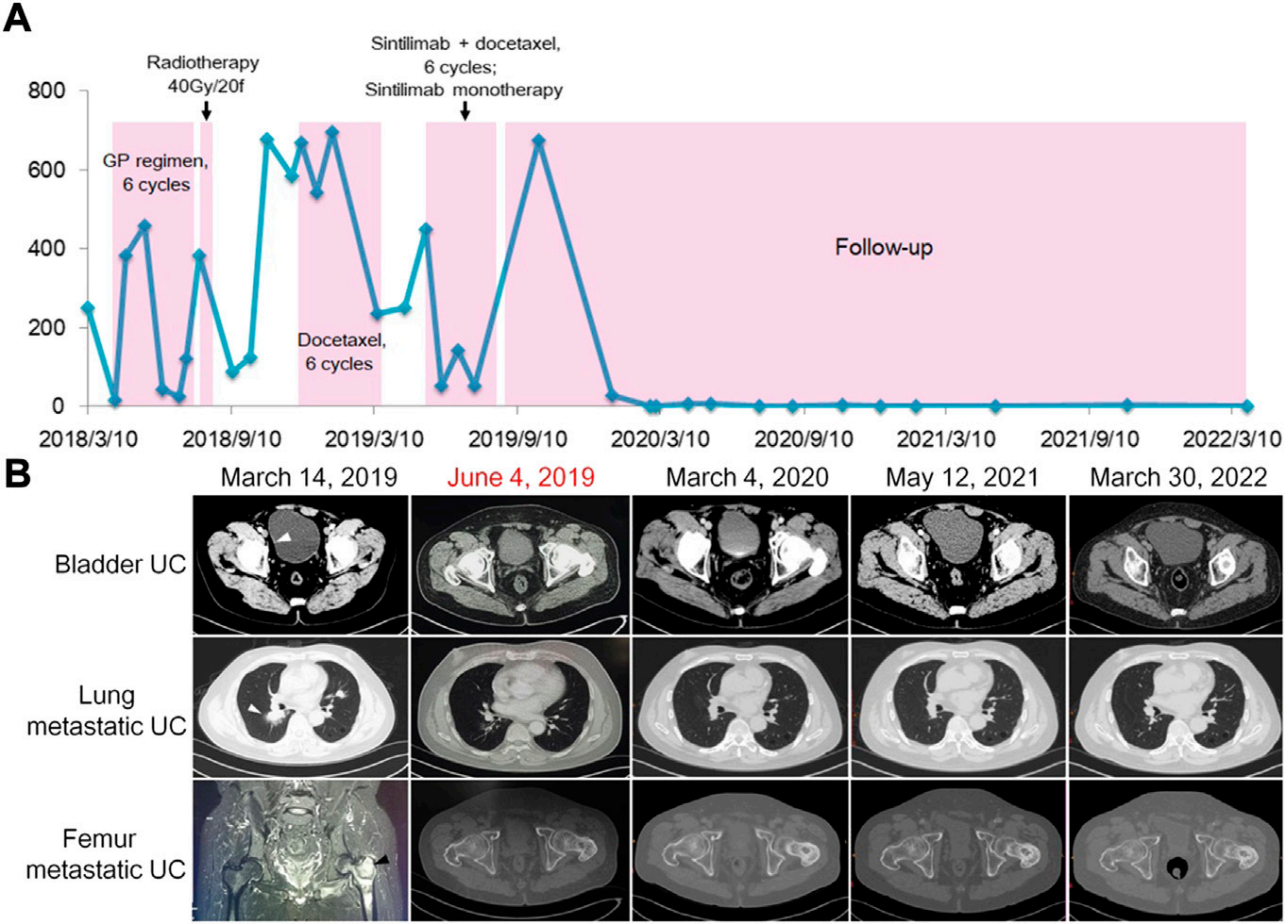
Clinical monitoring of treatment response in a patient with recurrent and metastatic UC. **(A)** Red blood cell count monitoring in urine. **(B)**. Images of bladder UC (arrowhead) and metastatic lesions (arrowheads) before and after combined immunotherapy, exhibiting vanishing and stabilization of tumors.

On 1 March 2018, computed tomography (CT) showed bilateral multiple pulmonary nodules, with needle biopsy and pathology demonstrating metastatic UC. A solid nodule in the right posterior bladder wall was considered recurrent UC. During the next 4 months, he received six cycles of gemcitabine (1 g/m^2^, 1.6 g on day 1) plus cisplatin (75 mg/m^2^, 40 mg on days 1, 2, and 3, every 21 days). Magnetic resonance imaging (MRI) showed multiple bone metastases in the left proximal femur, pelvis, and left lower extremity bone. The patient underwent radiotherapy (95% PTV, 40 Gy/20f) for bone metastasis over 4 weeks. He also received six consecutive cycles of docetaxel (75 mg/m^2^, 120 mg on day 1, every 21 days) between 9 December 2018 and 13 March 2019, during which urinalysis displayed consistently high red blood cell (RBC) counts (Figure 1A). Imaging on 14 March 2019 revealed progressive bladder UC and aggravated bilateral lung metastasis and left femur metastasis (Figure 1B). Treatment response evaluation identified stable disease according to the response evaluation criteria for solid tumors. Moreover, gene testing of UC showed a microsatellite instability–high (MSI-H) phenotype. Accordingly, the patient was subsequently started on four cycles of the PD-1 inhibitor sintilimab (200 mg on day 1) plus docetaxel (120 mg on day 2, every 21 days) on 22 April 2019. After cycle two, a CT scan on 4 June 2019 revealed a disappeared nodule in the bladder, lung, and left proximal femur (Figure 1B). The patient had pruritus and maculopapular rash along the head and neck following sintilimab injection. Loratadine was effective against the allergic symptoms. He received the last sintilimab (200 mg on day 1) monotherapy on 30 July 2019. Two months later, the patient was again admitted to the hospital because of long-term fever and anorexia. Laboratory tests showed a free thyroxine level of 42 pmol/L, thyroid stimulating hormone 0.01 μIU/ml. Adrenocorticotropic hormone was assessed, which was <1 pg/ml (7.2–63.4 pg/ml) and hydroxycorticosteroid level 1.4 mg/24 h (2.0–10.0 mg/24 h), a morning cortisol level 8.4 nmol/L (172–497 nmol/L). Accordingly, the patient was diagnosed with sintilimab-associated hypophysitis and hypothyroidism. He was treated with thiamazole (10 mg, daily) and prednisone (5 mg at 8:00 a.m. and 2.5 mg at 4:00 p.m.). The patient’s symptoms improved significantly. Thereafter, thyroxine (0.2 mg) and prednisone (7.5 mg) were taken orally every day. According to the Common Terminology Criteria for Adverse Events v5.0 (https://www.gbg.de/de/rechner/ctcae.php), the patient’s symptoms met the criteria of Grade 2. Between 2020 and 2022, urinary RBC count was tested every 3 months and CT imaging of the chest/pelvis/bones was done every 6 months. Up to 30 March 2022, the patient remained symptom-free. Urinalysis (Figure 1A) and CT imaging (Figure 1B) showed a sustained response to therapy.

### Immunohistochemistry and Gene Testing

**FIGURE 2:**
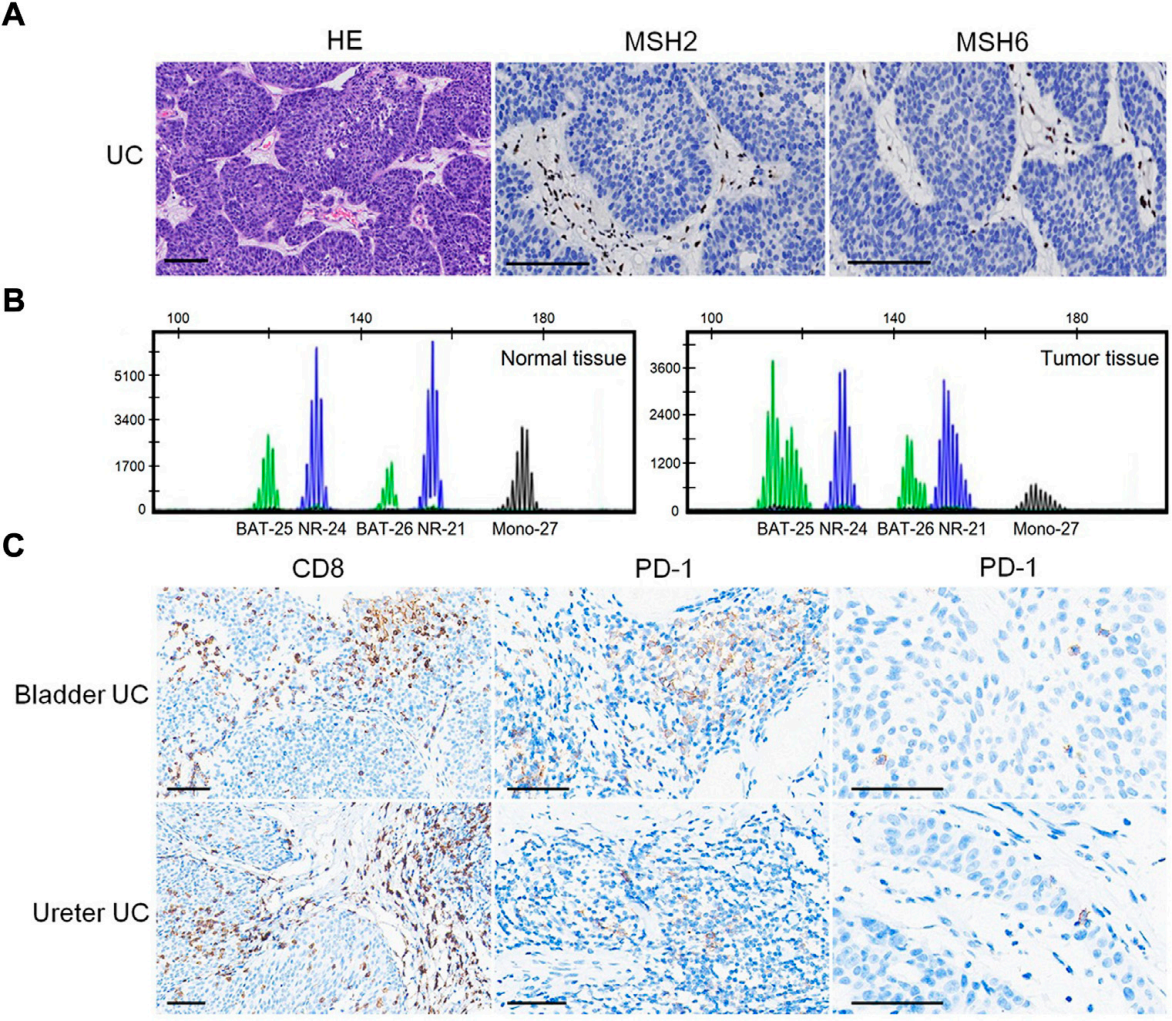
Predictive biomarkers for assessment of therapeutic response. **(A)** Loss of MSH2 and MSH6 expression in UC. Bar = 100 μm. **(B)** MSI analysis based on a pentaplex panel. **(C)** CD8 and PD-1 expression in UC. Bar = 50 μm.

The patient had multiple metachronous cancers, including stomach cancer at age 34 years, colon cancers at ages 40 and 54 years, and skin cancer at age 66 years. We detected the expression of MMR proteins (MLH1: clone ES05, 1:100, PMS2: clone EP51, 1:50, MSH2: clone RED2, 1:100, and MSH6: clone EP49, 1:50; DAKO) in tumors using immunohistochemistry (IHC) staining. As shown in Figure 2A, loss of MSH2 and MSH6 expression was observed in UC, suggesting a dMMR status in UC.

We performed MSI gene locus assays with a pentaplex panel of five mononucleotides (BAT-25, BAT-26, NR-21, NR-24, and MONO-27) using the Promega MSI Analysis System. The results showed four (BAT-25, BAT-26, NR-21, and MONO-27) loci with instability (Figure 2B), indicating an MSI-H status. IHC staining showed abundant CD8^+^ (clone SP16, 1:200; ZSGB-BIO, China) T-cell infiltration either at the tumor–stroma interface or within the tumor mass (Figure 2C). PD-1 (clone OTI4F10, 1:100, ZSGB-BIO) expression was identified mainly on immune cells (ICs) in the stroma of UC, as well as in scattered single tumor cells (TCs), with a combined positive score (CPS: number of PD-1 expressing cells TCs and ICs relative to the total number of TCs) = 8 (Figure 2C). However, no PD-L1 (clone 22C3, 1:250, DAKO) expression was identified on ICs or TCs (CPS = 0; figures not shown).

**FIGURE 3:**
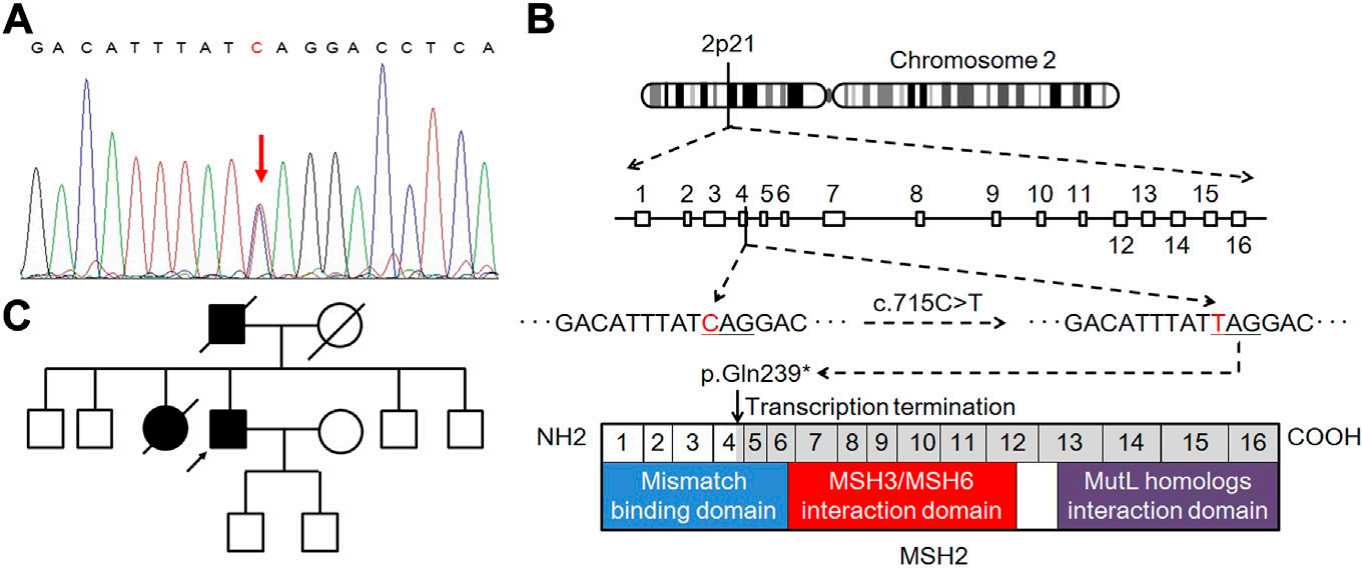
Genetic testing and pedigree analysis. **(A)** Heterozygous pathogenic germline mutation in the MSH2 gene of the proband. **(B)** Schematic representation of deleterious **C**.715C>T (*p*.Gln239*) mutation in MSH2 exon 4, leading to premature transcription termination and non-functional MSH2 protein product. **(C)** Pedigree chart of the family.

In genetic assessment using direct sequencing, we detected a heterozygous pathogenic germline mutation of c.715C>T (*p*.Gln239*) in *MSH2* exon 4 (Figure 3A), which resulted in a premature stop codon and caused a truncated or absent MSH2 protein (Figure 3B). Accordingly, the patient was diagnosed with LS [6]. The pedigree chart of the family is presented in Figure 3C.

## Discussion

Here, we report the case of a patient with recurrent and metastatic LS-associated UC who achieved a sustained response to PD-1 inhibitors combined with chemotherapy. Although the patient received cisplatin-based first-line chemotherapy and docetaxel second-line monotherapy, disease progression was detected, including local recurrence and lung/bone metastasis, suggesting limited benefit from chemotherapy. As a PD-1 inhibitor, sintilimab is currently approved in China but not in other countries for tumor immunotherapy. Sintilimab displays few adverse reactions and significant efficacy against a variety of solid tumors [7]. However, there are limited reports about the treatment of UC with PD-1 inhibitor therapy alone or combined with other chemotherapeutic drugs. A recent report demonstrated the partial response to sintilimab combined with nanoparticle albumin-bound-paclitaxel therapy for recurrent bladder UC and pelvic metastasis over an 11-month follow-up [8]. In the present report, our patient with recurrent and metastatic UC achieved sustained response after PD-1 inhibitor therapy combined with docetaxel therapy over 31 months. Hence, PD-1 inhibitor therapy combined with chemotherapy is a promising therapeutic strategy for patients with recurrent and metastatic UC whose disease progresses after first-line and second-line treatment.

UC is considered the third most common cancer in LS and is included in both the revised Bethesda Guidelines and the Amsterdam II criterion [9]. LS-associated upper tract urothelial cancer (UTUC) has a reported lifetime individual risk between 2.9% and 28%, conferring up to a 22-time greater risk in LS than in the general population [10]. The relative risk of bladder UC in LS is 12.3% for men and 2.6% for women, with MSH2 mutations and MSI-H seen in 86% and 85.7% of cases, respectively [11]. The present patient with a personal/familial history displayed germline MSH2 gene mutation, dMMR (MSH2/MSH6 deficiency), and MSI-H in UC. Accordingly, he was diagnosed with LS. dMMR in LS-associated tumors resulting from germline mutations of MMR genes increases the likelihood of acquiring somatic genetic mutations, particularly in short-tandem repeat sequences, leading to MSI. Previous case reports have also shown prolonged, complete remission at least 11 months in a patient with sporadic, high-grade dMMR UC of the renal pelvis, who received combined PD-L1 inhibitor immunotherapy [12]. Recently, clinical trials have confirmed the predictive value of dMMR/MSI status for PD-1-targeted inhibitor pembrolizumab therapy in patients with dMMR/MSI-H unresectable/metastatic CRC and advanced endometrial cancer [4, 5]. MSI-H has been detected in 21% of UTUC cases, 87% of which were dMMR [13]. The present patient with dMMR/MSI-H recurrent and metastatic UC obtained significant and durable clinical benefits from combination immunotherapy. These studies suggest the dMMR/MSI status in UC is a potential biomarker for predicting the response to immunotherapy. Moreover, dMMR/MSI status should be fully investigated in all UC cases, especially for patients who meet the Bethesda Guidelines and the Amsterdam II criteria.

Tumors with dMMR/MSI-H produce a plethora of immunogenic neoantigens, resulting in increased CD8^+^ lymphocyte infiltration and upregulation of genes encoding immune checkpoints, such as PD-1, PD-L1, and CTLA4 [14]. Although it is reported that dMMR is uncommon (2%) in muscle-invasive and high-grade UC of the bladder, the increased cytotoxic T lymphocytes and PD-L1 expression in these cases have been demonstrated [15]. Indeed, high *versus* low PD-L1 expression is associated with a greater likelihood of treatment benefit with anti-PD-L1 therapy in advanced or metastatic UC [16]. Companion PD-L1 IHC diagnostic assays are approved by the US Food and Drug Administration (FDA) for use in patients with advanced UC. In the present patient, PD-L1 expression not detected on TCs or on ICs. However, we observed more PD-1 expression on ICs than on TCs, which suggested the potential predictive value of PD-1 expression in the response to immunotherapy. However, larger studies are required to fully investigate PD-1 expression in all UC cases and treatment benefits from PD-1/PD-L1 blockade therapy. Also, scoring algorithms and validation metrics of PD-1 IHC tests require more research in the future.

In conclusion, we describe a patient with recurrent and metastatic LS-associated UC who achieved a sustained response to PD-1 inhibitors combined with chemotherapy over 31 months, during which the side effects of immunotherapy could be controlled and managed. Our case supports the inclusion of such combination and/or monotherapy in clinical studies and using dMMR/MSI-H status and PD-1 expression as potential predictive markers for the assessment of therapeutic response.

## Data Availability

The original contributions presented in the study are included in the article/supplementary material, further inquiries can be directed to the corresponding authors.
