# Corrigendum: Case report: Potential predictive value of MMR/MSI status and PD-1 expression in immunotherapy for urothelial carcinoma

**DOI:** 10.3389/pore.2023.1610989

**Published:** 2023-01-30

**Authors:** Yu-Ting Ma, Yan Li, Li Yan, Fang Hua, Dong-Guan Wang, Guo-Ying Xu, Hong-Lan Yang, Ying-Jie Xue, Ye-Jun Qin, Dan Sha, Hao Ning, Miao-Qing Zhao, Zhi-Gang Yao

**Affiliations:** ^1^ Department of Pathology, Shandong Provincial Hospital Affiliated to Shandong First Medical University, Jinan, China; ^2^ Departmentof Oncology, Dongying City People’s Hospital, Dongying, China; ^3^ Department of Pathology, Dongying City People’s Hospital, Dongying, China; ^4^ Department of Microbiology and Immunology, Tulane University, New Orleans, LA, United States; ^5^ Department of Urology Surgery, Dongying Hospital of Traditional Chinese Medicine, Dongying, China; ^6^ Department of Oncology, Shandong Provincial Hospital Affiliated to Shandong First Medical University, Jinan, China; ^7^ Department of Urology, Shandong Provincial Hospital Affiliated to Shandong First Medical University, Jinan, China; ^8^ Department of Pathology, Shandong Cancer Hospital and Institute, Shandong First Medical University, Shandong Academy of Medical Sciences, Jinan, China

**Keywords:** immunotherapy, microsatellite instability, immune checkpoint inhibitors, urothelial carcinoma, lynch syndrome, PD-1/PD-L1

In the published article there was an error in the author list. Authors Hong-Lan Yang and Yan Li were listed in the wrong order. The equal contribution of the first co-authors was not included. The corrected author list appears below:

Yu-Ting Ma^1‡^, Yan Li^2‡^, Li Yan^3‡^, Fang Hua^4^, Dong-Guan Wang^3^, Guo-Ying Xu^5^, Hong-Lan Yang^2^, Ying-Jie Xue^1^, Ye-Jun Qin^1^, Dan Sha^6^, Hao Ning^7^, Miao-Qing Zhao^8^* and Zhi-Gang Yao^1^*^†^

^†^ORCID: Zhi-Gang Yao, orcid.org/0000-0002-8034-6524

^‡^Contributed equally to this work

The authors apologize for these errors and state that they do not change the scientific conclusions of the article in any way. The original article has been updated.

